# Fine Mapping of Wheat Stripe Rust Resistance Gene *Yr26* Based on Collinearity of Wheat with *Brachypodium distachyon* and Rice

**DOI:** 10.1371/journal.pone.0057885

**Published:** 2013-03-05

**Authors:** Xiaojuan Zhang, Dejun Han, Qingdong Zeng, Yinghui Duan, Fengping Yuan, Jingdong Shi, Qilin Wang, Jianhui Wu, Lili Huang, Zhensheng Kang

**Affiliations:** 1 State Key Laboratory of Crop Stress Biology for Arid Areas and College of Plant Protection, Northwest A&F University, Yangling, Shaanxi, P. R. China; 2 State Key Laboratory of Crop Stress Biology for Arid Areas and College of Agronomy, Northwest A&F University, Yangling, Shaanxi, P. R. China; 3 State Key Laboratory of Crop Stress Biology for Arid Areas and College of Life Science, Northwest A&F University, Yangling, Shaanxi, P. R. China; Kansas State University, United States of America

## Abstract

The *Yr26* gene, conferring resistance to all currently important races of *Puccinia striiformis* f. sp. *tritici* (*Pst*) in China, was previously mapped to wheat chromosome deletion bin C-1BL-6-0.32 with low-density markers. In this study, collinearity of wheat to *Brachypodium distachyon* and rice was used to develop markers to saturate the chromosomal region containing the *Yr26* locus, and a total of 2,341 F_2_ plants and 551 F_2∶3_ progenies derived from Avocet S×92R137 were used to develop a fine map of *Yr26*. Wheat expressed sequence tags (ESTs) located in deletion bin C-1BL-6-0.32 were used to develop sequence tagged site (STS) markers. The EST-STS markers flanking *Yr26* were used to identify collinear regions of the rice and *B. distachyon* genomes. Wheat ESTs with significant similarities in the two collinear regions were selected to develop conserved markers for fine mapping of *Yr26*. Thirty-one markers were mapped to the *Yr26* region, and six of them cosegregated with the resistance gene. Marker orders were highly conserved between rice and *B. distachyon*, but some rearrangements were observed between rice and wheat. Two flanking markers (*CON-4* and *CON-12*) further narrowed the genomic region containing *Yr26* to a 1.92 Mb region in *B. distachyon* chromosome 3 and a 1.17 Mb region in rice chromosome 10, and two putative resistance gene analogs were identified in the collinear region of *B. distachyon*. The markers developed in this study provide a potential target site for further map-based cloning of *Yr26* and should be useful in marker assisted selection for pyramiding the gene with other resistance genes.

## Introduction

Wheat (*Triticum aestivum* L.) is an important crop and a primary food source for humans. Stripe rust, caused by the fungal pathogen *Puccinia striiformis* Westend. f. sp. *tritici* Erikss. (*Pst*), is an important disease of wheat in China and many other countries. To date, 53 stripe rust resistance genes (*Yr1*–*Yr53*) and numerous temporarily designated genes have been reported in wheat (http://wheat.pw.usda.gov/cgi-bin/graingenes). Most of these genes have been mapped on chromosomes and/or specific chromosomal regions, and many of them have been used in wheat breeding programs worldwide. However, with the spread of *Pst* race CYR32, a large number of known resistance genes are no longer effective in China [1].

Despite considerable progress in the identification and mapping of stripe rust resistance genes, only two adult plant resistance (APR) genes, *Yr18* [2] and *Yr36* [3], have been cloned. *Yr26* has been widely used in wheat breeding programs in China for developing stripe rust resistant cultivars [4], [5], varieties with *Yr26* are grown on more than 3.4 million hectares in China. As the gene is still effective against the current *Pst* populations, cloning *Yr26* is important for understanding the molecular mechanisms of resistance. The *Yr26* gene, which is present in the common wheat line 92R137, was derived from Chinese *T. turgidum* landrace γ80-1 [6]. The gene was previously mapped near the centromere region, putatively on the short arm of wheat chromosome1 B with SSR markers *Xgwm11*, *Xgwm18* and *Xgwm413* [6]. A recent study located *Yr26* to the deletion bin C-1BL-6-0.32 with molecular markers *WE173* and *Xbarc181* [7]. The genetic distances between *Yr26* and the two closest flanking markers were 1.4 and 4.3 cM, respectively. Although several markers have been mapped to the *Yr26* region, the number of the markers is still limited, and more are needed for more efficient marker-assisted selection, fine mapping and map-based cloning of *Yr26*.

A perception is that fine mapping and map-based cloning in hexaploid wheat (*T. aestivum*, 2n = 6x = 42, genomic formula AABBDD) faces enormous challenges because of the huge genome size (17 Gb), polyploidy and highly repetitive sequences (>80%) within the genome. This problem can be solved, at least partially, by leveraging the physically mapped wheat ESTs [8], [9] and conserved syntenic relationship between wheat and model grass species [10], [11], [12], [13]. Collinearity of chromosome regions between wheat and model species, such as rice and *B. distachyon*, is well characterized [9], [14]. The available whole genomic sequences of rice and *B. distachyon* provide useful information for developing molecular markers, identifying candidate genes for traits of interest, predicting biological functions of genes and cloning genes. Such comparative genomic approaches have been used in map-based cloning of many wheat genes, including *Yr18* [2] and *Yr36* [3] for stripe rust resistance, and vernalization response genes *Vrn1*, *Vrn2* and *Vrn3* [15], [16], [17]. A particular challenge to map-based cloning of *Yr26* is its proximity to the centromere and that small recombination distances in such regions may correspond to huge physical distances at the DNA level.

Towards fine mapping and map-based cloning of *Yr26*, the objective of this study was to saturate the chromosome region containing *Yr26* through comparative genomics analysis using genomic sequences of rice and *B. distachyon* and available wheat ESTs. Markers closely linked to *Yr26* should be useful for marker-assisted selection and contribute towards map-based cloning of this gene.

## Results

### Genetic Analysis of Stripe Rust Response

Seedlings of 92R137 were resistant (IT 0;) and those of AvS were susceptible (IT 4) in a seedling test with race CYR32. The F_2_ population segregated in 1,747 resistant and 594 susceptible, fitting a 3∶1 ratio (χ^2^_3∶1_ = 0.17, *P* = 0.68), indicating that *Yr26* in the AvS×92R137 population behaved as a single dominant gene. Among the 551 F_2∶3_ families tested with the same race, 147 of 409 families derived from resistant F_2_ plants were homozygous resistant, 262 segregated and 142 families derived from susceptible F_2_ plants were homozygous susceptible. The segregation of these families conformed to a 1∶2: 1 ratio (χ^2^_1∶2:1_ = 1.36, *P = *0.51) as expected for a single gene.

The 92 F_2∶3_ lines recombinant between markers *WE201* and *STS-BQ6* were further tested with CYR32 to verify their phenotypes. The responses were consistent with earlier F_2_ phenotypes; that is, 49 F_2∶3_ families derived from susceptible F_2_ plants were homozygous susceptible, 7 of 43 families derived from resistant F_2_ plants were homozygous resistant and 36 were segregating. The results from the recombinant evaluations indicated that the phenotypes of the F_2_ plants were accurately classified.

### Development of *Yr26*-linked EST-STS Markers from Wheat ESTs

**Figure 1:**
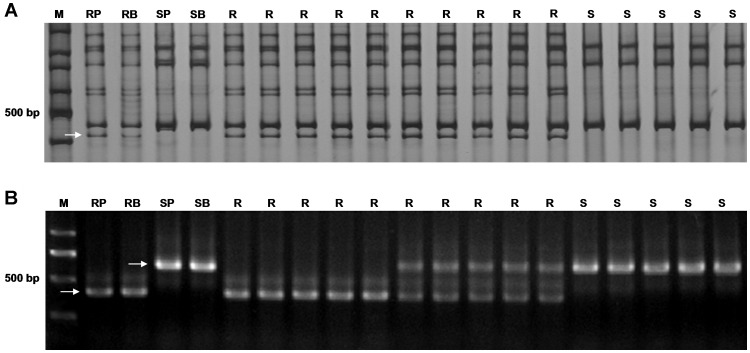
PCR amplifications of the markers on partial plants of the F_2_ population. A: dominant marker *STS-BQ33*; B: codominant marker *STS-CD77*; RP, 92R137; RB, resistant bulk; SP, AVS; SB, susceptible bulk; R, resistant plants; S, susceptible plants; M, 100 bp marker (A) and D 2000 (B); Arrows indicate the polymorphic bands.

Six EST-STS markers (*WE173*, *WE171*, *WE177*, *WE201*, *WE202* and *WE210*) linked with *Yr26* in an F_2_ population of 92R137×Yangmai 5 [7] were tested for polymorphism in cross AvS×92R137. Only *WE173* and *WE201* showed clear polymorphisms between the parents and bulks. Of 163 newly developed EST-STS markers, eight (*STS-BQ5*, *STS-BQ6*, *STS-CD28*, *STS-BQ33*, *STS-BE46*, *STS-BE68*, *STS-BQ74* and *STS-CD77*) produced stable polymorphic bands in the bulk segregant analysis. Among the 10 polymorphic EST-STS markers, 4 (*STS-BQ5*, *STS-BQ33* and *STS-BE46*) were dominant and 7 were codominant (examples shown in Figure 1). The codominant markers *STS-CD77* and *WE173* were detected using both agarose gel and polyacrylamide gel electrophoresis. All ten EST-STS markers (Table 1) were used to genotype the entire F_2_ population of 2,341 plants.

**Table 1:** Molecular markers mapped at or close to the *Yr26* locus.

Marker	Wheat EST	Forward primer (5′-3′)	Reverse primer (5′-3′)	Annealing Temperature (°C)
*STS-BQ5* ^a^	BQ160738	TCCTGACACCAAAGTAACCG	ATAGCCAAGCCCCATTCC	52
*STS-BQ6* ^a^	BQ165938	GAAAAGGGTACAATGATGAGTG	CCAGCAGAAACAAAAAAAGG	53
*STS-CD28* ^a^	CD453471	ACTACTCTTTATTCGTCCCAAC	TCGTCTCTGATGACCACAAC	52
*STS-BQ33* ^a^	BQ160383	TAAACCAAGTCCCCCAAA	GGAGTCCATCTTCACCGA	55
*STS-BE46* ^a^	BE493918	CCGTACTACAGCTACTCGC	CATCGTTCAGGTAATCGTC	51
*STS-BE68* ^a^	BE443531	GAGGTAGATAACACTGATGCG	CATAACTTCTCTCCCGACAC	52
*STS-BQ74* ^a^	BQ169964	TGGATGAACCAACGATAGT	TGGGAAACACTTGACTGC	53
*STS-CD77* ^a^	CD490549	CGACGAAGCCGTTGTTAT	TCAAGCAAAGACGAGAGGAT	50
*WE201* ^a^	BE497109	GCCTGCGAAACTCAGAATGT	CCAAAGCAAATGCCACAGTA	54
*WE173* ^a^	BF474347	GGGACAAGGGGAGTTGAAGC	GAGAGTTCCAAGCAGAACAC	55
*CON-1* b	DR741860	CGCAACAGTTCAACCATACA	ATCCTGCTCAGACCCAAAG	61
*CON-2* b	CJ729769	GTTGGATTTGTCGGTGAA	TCTGAGCGATGTAATGGTG	55
*CON-3* b	DR741641	GGCGGAAACCACGAGACC	CGGCGAGATGGAGCGACT	55
*CON-4* b	CJ883804	GTGCTGTACCTGACGACGGA	GTGGAGATGTTGGGCTTGG	58
*CON-5* b	CD936328	GTGACATCAAGCCAGACAACT	GAATCTCAGGGAACGACAATA	52
*CON-6* b	CD939050	GCCGATGGGGAACTGAAT	GTTGAACCGCTTGAACACC	53
*CON-7* b	CJ955255	CGGCTCCCAAAGGAAGAAT	AGGGGAGTCACTTTATGGATTTT	58
*CON-8* b	GH728673	TTGGAAGTGTACCCGTGAG	AGGGCATTTACTGCTGTGAG	55
*CON-9* b	CJ954892	GGCAGTAGCCAGGGCAAGA	CCAAGCTGCGCCCATGTAA	60
*CON-10* b	CJ550732	ATACTTCAGGAAAATGTTCGA	TTTATTAGGTTGCTTTAGGG	52
*CON-11* b	CA744306	TAGCCTTGACAAGTTCCTCT	GTATCATTGATTTTCCGAC	50
*CON-12* b	BJ280972	CAGTGGACGGAAAGAAGTG	TAGCAGTCAAAGTGGGAGC	53
*CON-13* b	CJ663781	GAACAGAGGCGAAGGCAGGA	AGCGGGTGGAAGCCGTAGT	52
*CON-14* b	BQ246252	GCTTCAGCAGTTACCACATAC	TACCTTCATCCAGCATCATC	50
*CON-15* b	CJ704659	GTAAACGGTTGTCAGACGG	GTTCAGAACTAGCGATGCC	59
*CON-16* b	CF133841	CGTCTACAGGTTCGACAAC	TCTTACGCTTCTTAGGGTTT	56
*CON-17* b	CJ831661	GGTATTCGCAGGCAACTCA	ACATCACCTCCCACAGGCT	52
*CON-18* b	CJ675116	ACCCCGACGGCCTTCAACT	ACGATGGTGCCGAAGAGCA	62
*CON-19* b	CJ805435	AAAATTGTACCACCAGATTG	TTTGAAGCCTGTGAGAAAA	52
*CON-20* b	GH723446	ACGCTGCTGCTGGTGTCGT	TCCAGGATGTAGGGGTCGC	60
*CON-21* b	CJ803731	TTATTGTCGGCTGAACCAG	GCCAGGGATGAGCTTTTAT	53

a, b Marker types: STS marker derived directly from wheat EST,

b Conserved marker developed by comparative analysis of wheat with *B. distachyon* and rice, and designed using Conserved Primers 2.0.

### Development of Conserved Markers through Comparative Genomics of Wheat with *B. distachyon* and Rice

**Figure 2:**
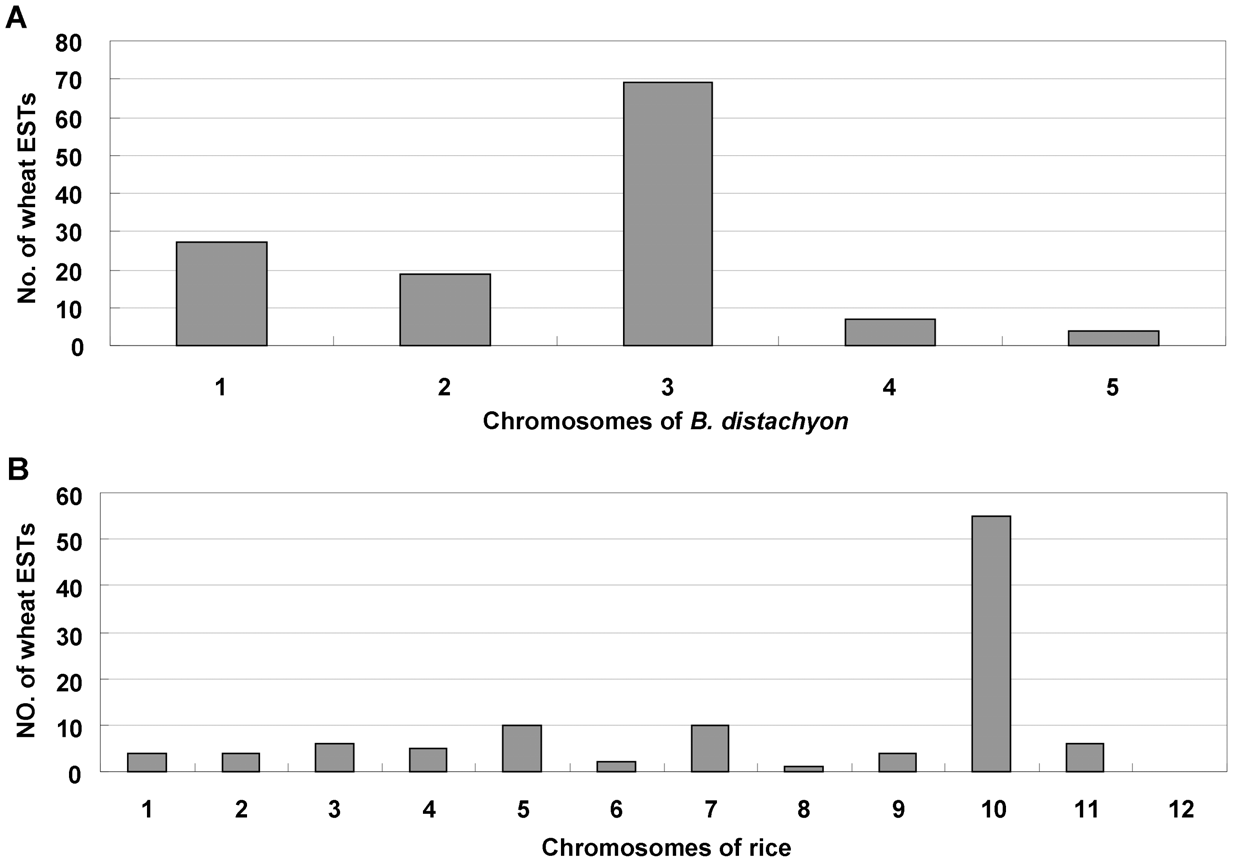
Frequency distributions of wheat ESTs related to *B.distachyon* and rice genes.

To develop more markers for *Yr26*, 169 wheat ESTs in deletion bin C-1BL-6-0.32 were used to identify their similar genomic sequences in *B. distachyon* and rice; 126 had significant similarities to *B. distachyon* sequences and 107 had similar sequences in rice. The distributions of these similar ESTs on chromosomes of *B. distachyon* and rice are shown in Figure 2. Sixty-eight of 126 ESTs were located on *B. distachyon* chromosome 3 and 56 of 107 ESTs were closely related to sequences on rice chromosome 10. The results indicated a synteny between wheat chromosomal bin C-1BL-6-0.32, *B. distachyon* chromosome 3 and rice chromosome 10.

To accurately characterize the collinearity between the *Yr26* region and the genomic regions of *B. distachyon* and rice, ten sequences corresponding to the mapped wheat ESTs were used as queries to perform a BLAST search against the rice and *B. distachyon* genome sequences. Orthologs of four wheat ESTs, BQ165938, CD453471, BQ160383 and BE443531, were detected on *B. distachyon* chromosome 3, and the first three were detected on rice chromosome 10. The other six wheat ESTs, BQ160738, BE493918, BQ169964, CD490549, BE497109 and BF474347, either had significant similarities to sequences on other chromosomes of *B. distachyon* and rice, or the scores and E values were not in accordance with the search parameters (Table S1). Comparative genomic analysis established the collinearity of the *Yr26* genomic region with a 4.48 Mb region (*Bradi3g28070* – *Bradi3g31630*) in *B. distachyon* chromosome 3 and a 3.33 Mb region (*Os10g0462900* – *Os10g0524500*) in rice chromosome 10. The collinear regions in rice and *B. distachyon* were covered by the EST-STS markers *STS-CD28* and *STS-BQ33*, and the *Yr26* region was therefore identified to be syntenic to parts of *B. distachyon* chromosome 3 and rice chromosome 10.

**Figure 3:**
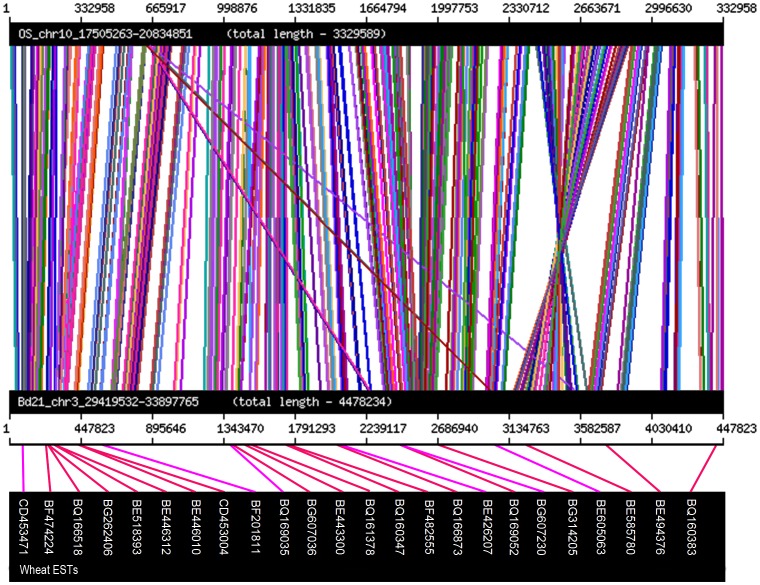
Collinearity between 4.48 Mb region of *B. distachyon* and 3.33 Mb region of rice and wheat ESTs. 4.48 Mb: *Bd3g28070 – Bd3g31630*; 3.33 Mb: *Os10g0462900 – Os10g0524500*; 24 wheat ESTs; The different colors showed the scores in the BLASTNn. Black, ≤40; Blue, 40–50; Green, 50–80; Purple, 80–200; Red, ≥200.

There are 328 genes in the 4.48 Mb region of *B. distachyon* and 237 genes in the 3.33 Mb region of rice. After alignment of all of the genes present in the collinear regions of rice and *B. distachyon*, 207 *B. distachyon* genes had significant similarities with the corresponding rice interval and 191 rice genes had similar DNA sequences in the collinear *B. distachyon* region. The relationship between wheat ESTs, the rice and *B. distachyon* genes located in the collinear regions was displayed using the Artemis Comparison Tool [30]. As shown in Figure 3, most gene orders were conserved, but there were some rearrangements. Genes located in the collinear regions of *B. distachyon* and rice were used as queries to search for orthologous wheat ESTs in the wheat EST database (http://wheat.pw.usda.gov/GG2/blast.shtml) and the identified wheat ESTs were used to design primers. A total of 358 conserved primers were designed using Conserved Primers 2.0 [26] and used to determine polymorphisms between the parents and bulks. Twenty one conserved markers were found to be polymorphic (Table 1). Among the 21 conserved polymorphic markers (Table 1) most, such as *CON-3*, *CON-6*, *CON-8* and *CON-11*, were codominant (Figure. S1). All 21 conserved markers were used to genotype the 43 recombinants between *STS-CD28* and *STS-BQ33*.

### High Resolution Map for *Yr26* and Collinearity Relationships of Wheat EST Markers with Orthologs in *B. distachyon* and Rice

**Figure 4:**
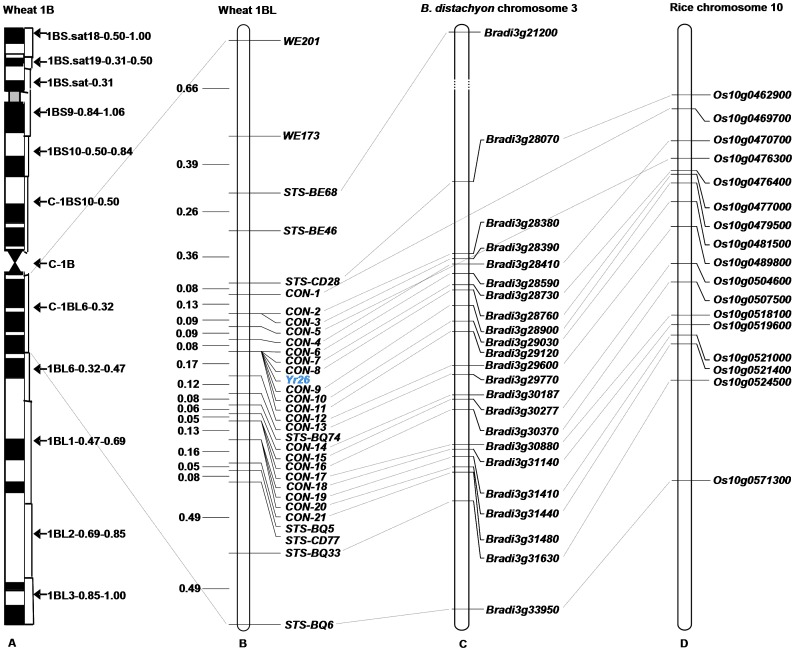
Physical and genetic maps for stripe rust resistance gene *Yr26* corresponding to comparative genomic maps of *B. distachyon* and rice. A: physical map of wheat 1B; B: genetic map of *Yr26*; C: *B. Distachyon* chromosome 3; D: rice chromosome 10; Marker names are indicated on the right side of the map. Map distances (cM) are shown on the left side. Collinear genes are indicated to the right of *B. distachyon* chromosome 3 and rice chromosome 10 based on chromosome Mb positions.

A high resolution map for *Yr26* in deletion bin C-1BL-6-0.32 (Figure 4A) was constructed with 31 markers, including the 10 EST-STS markers developed directly from wheat ESTs and 21 conserved markers developed through synteny analysis with *B. distachyon* and rice (Figure 4B, C, D; Table 1). The ten EST-STS markers were closely linked to *Yr26* with genetic distances ranging from 0.43 to 2.14 cM (Table 2). The conserved markers, which further greatly saturated the linkage map (Figure 4B), were found to be closely linked with the *Yr26* locus and fell within a genetic interval of 1.16 cM (0.39 and 0.77 cM on two sides of the gene), and six of them, *CON-6*, *CON-7*, *CON-8*, *CON-9*, *CON-10* and *CON-11*, cosegregated with *Yr26*. Two conserved markers, *CON-4* and *CON-12*, flanked the *Yr26* locus at genetic distances of 0.08 and 0.17 cM (Figure 4B).

**Table 2:** Genetic linkages between *Yr26* and 10 polymorphic EST-STS markers in AvS×92R137.

Marker	R plants			S plants			Expected ratio	χ^2^	Distance from *Yr26* (cM)^a^
	A	H	B	A	H	B			
*STS-BQ5*	1740	–	7	10	–	584	A:B = 3∶1	0.08	0.82
*STS-BQ6*	560	1169	18	3	22	569	A:H:B = 1∶2:1	1.09	1.88
*STS-CD28*	570	1171	6	0	5	589	A:H:B = 1∶2:1	0.57	0.47
*STS-BQ33*	1737	–	10	22	–	572	A:B = 3∶1	0.02	1.39
*STS-BE46*	1737	–	10	9	–	585	A:B = 3∶1	0.23	0.83
*STS-BE68*	568	1166	13	1	11	582	A:H:B = 1∶2:1	0.64	1.09
*STS-BQ74*	567	1176	4	0	6	588	A:H:B = 1∶2:1	0.76	0.43
*STS-CD77*	563	1175	9	1	10	583	A:H:B = 1∶2:1	1.03	0.90
*WE173*	564	1156	18	1	15	578	A:H:B = 1∶2:1	0.89	1.48
*WE210*	561	1161	25	3	21	570	A:H:B = 1∶2:1	1.04	2.14

For codominant markers: A = homozygous for the marker allele in resistant plants, B = homozygous for the marker allele in susceptible plants, H = heterozygous for the marker; for dominant markers: A = marker present; B = marker absent;

a Distances were estimated by JOINMAP version 4.0.

Comparative genomic analysis revealed that 23 and 17 wheat ESTs had similarities on *B. distachyon* chromosome 3 and rice chromosome 10, respectively (Figure 4C, D; Table 3), again revealing high levels of collinearity of the *Yr26* region with *B. distachyon* chromosome 3 and rice chromosome 10 (Figure 4B, C, D). The orders of these markers were highly conserved between wheat and *B. distachyon*, but there was a rearrangement between wheat and rice. The rearrangement was observed between markers *CON-5* (CD936328) and *CON-4* (CJ883804) (Figure 4B, D). The two most closely linked markers *CON-4* (CJ883804) and *CON-12* (BJ280972) narrowed the genomic region carrying *Yr26* to a 1.92 Mb (*Bradi3g28410* – *Bradi3g29600*) on *B. distachyon* chromosome 3 and 1.17 Mb (*Os10g0470700* – *Os10g0489800*) on rice chromosome 10. There are 135 and 68 genes in the narrowed collinear regions of *B. distachyon* and rice, respectively. No typical NBS-LRR resistance gene analog was found in the collinear regions of rice (*Os10g0470700 – Os10g0489800*) and *B. distachyon* (*Bradi3g28410* – *Bradi3g29600*). However, *Bradi3g28590* was annotated as “leucine-rich repeat (LRR) protein kinase”, and *Bradi3g29120* was annotated as “protein kinase”. The relationships between the putative LRR and protein kinase genes and *Yr26* need to be examined in more detail.

**Table 3:** Wheat ESTs corresponding to EST-STS markers and conserved markers, and similarity to *B. distachyon* and rice genomic sequences.

Wheat EST	*B. distachyon*			Rice		
	Gene	E value^a^	Position	Gene	E value^b^	Position
DR741860	na^c^	ns^d^	na	*Os10g0469700*	2e-101	17822238
CJ729769	*Bradi3g28380*	0	29713444	na	ns	na
DR741641	*Bradi3g28390*	0	29740626	na	ns	na
CJ883804	*Bradi3g28410*	0	29765151	*Os10g0470700*	0	17872818
CD936328	na	ns	na	*Os10g0476300*	4e-154	18196432
CD939050	*Bradi3g28590*	1e-94	29993923	na	ns	na
CJ955255	*Bradi3g28730*	0	30153785	*Os10g0476400*	0	18204024
GH728673	*Bradi3g28760*	0	30193649	*Os10g047700*	0	18235714
CJ954892	*Bradi3g28900*	6e-146	30666893	*Os10g0479500*	0	18458067
CJ550732	*Bradi3g29030*	1e-28	30875417	*Os10g0481500*	6e-32	18626119
CA744306	*Bradi3g29120*	4e-174	31031552	na	ns	na
BJ280972	*Bradi3g29600*	8e-135	31689499	*Os10g0489800*	3e-119	19045478
CJ663781	*Bradi3g29770*	2e-177	31808164	na		na
BQ246252	*Bradi3g30187*	0	32237291	na	ns	na
CJ704659	*Bradi3g30277*	0	32330105	*Os10g0504600*	0	19733671
CF133841	*Bradi3g30370*	4e-170	32457204	*Os10g0507500*	1e-86	19868554
CJ831661	*Bradi3g30880*	0	33133705	*Os10g0518100*	0	20480254
CJ675116	*Bradi3g31140*	0	33293299	*Os10g0519600*	0	20547000
CJ805435	*Bradi3g31410*	4e-143	33539338	*Os10g0521000*	3e-120	20609894
GH723446	*Bradi3g31440*	5e-107	33602254	*Os10g0521400*	1e-107	20630918
CJ803731	*Bradi3g31480*	0	33625009	na	ns	na
BQ165938	*Bradi3g33950*	4e-22	36391235	*Os10g0571300*	6e-13	23105285
CD453471	*Bradi3g28070*	4e-39	29414494	*Os10g0462900*	6e-24	17505263
BQ160383	*Bradi3g31630*	1e-140	33895891	*Os10g0524500*	1e-143	20832477
BE443531	*Bradi3g21200*	9e-67	20196538	na	ns	na

a E values in BLASTn between wheat EST and *B. distachyon* gene.

b E values in BLASTn between wheat EST and rice gene.

c na, not applicable.

d ns, not significant.

### PCR-based Markers for Marker-assisted Selection of *Yr26*

The 31 markers, including 25 closely-linked markers and 6 cosegregated markers, were used to test wheat cultivars/lines (Table 4) and to assess their potential in marker-assisted selection for *Yr26*. The results indicated that 11 markers (*STS-BQ33*, *STS-CD77*, *STS-BQ74*, *WE173*, *CON-1*, *CON-3*, *CON-4*, *CON-5*, *CON-6*, *CON-10* and *CON-19*) could be useful in selection of *Yr26* in breeding programs.

**Table 4:** Presence (+) and absence (−) of 11 molecular markers that can distinguish *Yr26* from other *Yr* genes in wheat genotypes.

Wheat genotype	Gene	*STS-BQ33*	*STS-BQ74*	*STS-CD77*	*WE173*	*CON-1*	*CON-3*	*CON-4*	*CON-5*	*CON-6*	*CON-10*	*CON-19*
AvS*Yr1*NIL^a^	*Yr1*	−d	−	−	−	−	−	−	−	−	−	−
AvS*Yr24*NIL	*Yr24*	−	+	+	+	+	+	+	+	+	+	+
AvS*Yr26*NIL	*Yr26*	+	+	+	+	+	+	+	+	+	+	+
Chuanmai 42	*YrCH42*	−	+	+	+	+	+	+	+	+	+	+
92R137^b^	*Yr26*	+	+	+	+	+	+	+	+	+	+	+
Chinese166^c^	*Yr1*	−	−	−	−	−	−	−	−	−	−	−
AvS		−	−	−	−	−	−	−	−	−	−	−

a The same pattern occurred for Avocet NILs possessing *Yr5*, *Yr6*, *Yr7*, *Yr8*, *Yr9*, *Yr10*, *Yr15*, *Yr17*, *Yr18* and *Yr27.*

b The same pattern occurred for 13 additional Chinese varieties with *Yr26* (Shanmai 107, Shanmai 175, Shanmai 139, Mianmai 39, Mianmai 42, Mianmai 96-5, Lantian 17, Zhong G918, Neimai 8, Neimai 9, Neimai 11, Neimai 836 and Chuannong 22).

c The same pattern occurred for 13 wheat varieties with known stripe rust resistance genes [Chinese 166 (*Yr1*), *Triticum spelta album* (*Yr5*), Mianyang 90–310/M 180 (*Yr6*), 8718/Chuanyu 12 (*Yr7*), Han 4599 (*Yr9*), Moro (*Yr10*), G-25 (*Yr15*), Chinese Spring (*Yr18*), Mian 2000–18 (*Yr27*), W7984 (*Yr28*), RSL65 (*Yr36*), Line 03524 (*Yr38*) and Chuannong 19 (*Yr41*)].

d ‘−’, same bands as AvS; ‘+’, same bands as AvS*Yr26* NIL and 92R137.

## Discussion

Despite the increasing numbers of stripe rust resistance genes identified and deployed in wheat breeding programs, only two have been cloned and characterized [2], [3]. In the present study we established a high resolution map of *Yr26* using a comparative genomics approach to provide a sound basis for further progress in map-based cloning of this gene.

There is collinearity among wheat chromosome 1B, rice chromosome 10 and *B. distachyon* chromosome 3 [9], [14], [31]. In the present study, most of the 169 wheat ESTs in the deletion bin C-1BL-6-0.32 were found to have significant similarities with genes on *B. distachyon* chromosome 3 and rice chromosome 10, confirming a close syntenic relationship and indicating that the genomic sequences of *B. distachyon* and rice should be useful for comparative analysis wheat genes. Rice was the first selected grass species for genome sequencing [32], [33] and *B. distachyon* is considered as the best model for wheat at present [34], [35]. In the present study, we found a higher number of orthologs between wheat and *B. distachyon* than between wheat and rice. This is consistent with the relationships among the three species as reported in the above studies.

Nevertheless, many exceptions to collinearity were observed in the comparisons of wheat, rice and *B. distachyon* due to rearrangements involving gene transposition, duplication, deletion and inversion [13], [36], [37]. Such anomalies in collinearity complicated the use of model species for genetics. These model grass genomes may not always provide sequence information to assist in identification of candidate gene. In the present study, gene deletions were observed when comparing the orthologous regions of rice and *B. distachyon*, in the collinear regions of rice (*Os10g0462900* – *Os10g0524500*) and *B. distachyon* (*Bradi3g28070* – *Bradi3g31630*), 46 rice genes had no orthologs in the corresponding region of the *B. distachyon* genome, and 121 genes predicted in *B. distachyon* had no orthologs in the corresponding region of the rice genome. Within the narrowed collinear regions between markers *CON-4* and *CON-12*, only two genes, *Bradi3g28590* and *Bradi3g29120* annotated as LRR and protein kinases, were present in the 1.92Mb region (*Bradi3g28410* – *Bradi3g29600*) of *B. distachyon*, but these were absent in the collinear region of rice (*Os10g0470700* – *Os10g0489800*).

Even if a target gene has no orthlogs in rice and *B. distachyon*, the flanking genes in those species are sufficiently conserved to provide useful information for developing conserved markers to saturate the target gene region in wheat. With 25 wheat genes found to have orthologs in *B. distachyon* and rice and six cosegregated markers for *Yr26*, the present study demonstrates a comparative genomics approach using the *B. distachyon* and rice sequences is effective for identifying markers for genes in wheat.

High resolution physical maps of wheat chromosomes showed that most disease resistance genes are arranged in clusters and are present mainly in the distal parts of the chromosomes [38]. Resistance genes cloned by map-based cloning, such as leaf rust resistance genes *Lr21* [39] and stripe rust resistance gene *Yr36* [3], are all distally located. In contrast, the target gene *Yr26* in this study maps to deletion bin C-1BL-6-0.32, a region that is near the centromere of chromosome 1B. Because recombination is limited around the centromere regions, with a consequent inflation in physical/genetic distances [40], map-based cloning of *Yr26* will be extremely difficult. However, we believe the difficulty can be overcome by integrating comparative genomics with BAC based chromosome walking torward the gene. The cosegregating and closely linked markers identified in the present study will be useful for screening the BAC library and identify BAC clones containing *Yr26*. Based on the high level of effectiveness and some evidence of race specificity, we hypothesize that *Yr26* could be a NBS-LRR type gene. The resistance gene candidates of the NBS-LRR type can be tested for resistance functions using gene silencing, mutation and transformation.

Cloning of the *Yr26* gene may contribute to understanding the mechanism of resistance at the molecular level and to a better understanding of this gene and its possible alleles. New markers developed in this study are diagnostic for *Yr26* and should facilitate rapid detection of *Yr26* (and putative alleles) in wheat cultivars and breeding lines, and therefore, can be used for pyramiding *Yr26* with other resistance genes to develop wheat cultivars with durable resistance.

## Materials and Methods

### Wheat Genotypes and Evaluation of Stripe Rust Reactions

A F_2_ population of 2,341 plants and 551 F_3_ line progenies with 30–40 plants in each, derived from a cross between susceptible genotype Avocet S (AvS) and resistant line 92R137 (*Yr26*), were used for genetic analysis and fine mapping of *Yr26*. For 92 F_2_ plants identified as recombinants between markers *WE201* and *STS-BQ6* flanking *Yr26* [7], 30–40 plants in each of their F_2∶3_ families were tested with *Pst* race CYR32 to confirm the phenotypes of the corresponding F_2_ plants. A total of 41 wheat genotypes were used to validate the molecular markers identified to be linked to the *Yr26* locus, including 13 *Yr* near-isogenic lines (NILs) of Avocet S (AvS), 13 Chinese wheat cultivars with *Yr26*, 13 wheat genotypes with known *Yr* genes and 2 genotypes (92R137 and AvS) as positive and negative controls for the *Yr26* allele (Table 4).

A predominant Chinese *Pst* race CYR32, which was avirulent on the AvS*Yr26* NIL and virulent on AvS, was used to test the F_2_ and F_2∶3_ populations and their parents. Seedlings grown in the greenhouse under controlled conditions were inoculated with fresh urediniospores when second leaves were fully expanded. Inoculated plants were incubated at 9°C and 100% relative humidity for 24 h and then transferred into a greenhouse with 14 h light (22,000 lx) at 17°C and 10 h of darkness at 12°C. Infection types (IT) were scored on a 0–4 scale [18] 15 days after inoculation when stripe rust symptoms were fully developed on the susceptible parent.

### DNA Extraction and Bulked Segregant Analysis

Genomic DNA was extracted from F_2_ seedlings of cross 92R137×AvS and the wheat genotypes described above using the sodium lauroylsarcosine protocol [19], [20]. Based on stripe rust response phenotypes, 10 resistant and 10 susceptible F_2_ plants with the same infection types as the resistant (IT 0;) and susceptible (IT 4) parents were selected to establish the resistant (BR) and susceptible (BS) bulks for bulked segregant analysis [21].

### Development of EST-STS Markers

Because *Yr26* was previously assigned to wheat chromosome deletion bin C-1BL-6-0.32 with six EST-STS markers (*WE201*, *WE202*, *WE210*, *WE171, WE173* and *WE177*) [7], these markers were used to test for polymorphisms between the present parents and bulks. In addition to those markers, 163 new pairs of EST-STS primers were designed from wheat ESTs mapped in the deletion bin (http://www.wheat.pw.usda.gov/index.shtml) using Primer Premier 5 software, and used in the bulked segregant analysis.

### Comparative Genomic Analysis and Conserved Marker Development

To develop more markers for *Yr26*, a comparative genomics approach was used. First, all of the 169 wheat ESTs assigned to deletion bin C-1BL-6-0.32 were used in BLASTn searching to identify collinear regions in the genomes of *B. distachyon* (http://www.brachypodium.org/) and rice (http://rice.plantbiology.msu.edu/cgi-bin/gbrowse/rice/). Homologous sequences of B. distachyon and rice were selected using an expected value of 1E^−10^ and identity ≧80% as cutoff points. Then ten mapped ESTs sequences were selected to identify collinear regions between the *Yr26* region, *B. distachyon* and rice based on the BLASTn results. The genes of *B. distachyon* and rice located in the collinear regions were used as queries to search the wheat EST database (http://wheat.pw.usda.gov/GG2/blast.shtml) using BLASTn. A total of 358 wheat ESTs were identified and used to design conserved markers [22], [23], [24], [25] using Conserved Primers 2.0 software [25].

### PCR Amplification and Electrophoresis

PCR was performed in a S1000 Thermal Cycler (BIO-RAD) for each DNA sample in a volume of 15 µl containing 1.0 U *Taq* DNA polymerase, 1.5 µl of 10× buffer (50 mmol KCl, 10 mmol Tris-HCl, pH 8.3), 2.0 mmol MgCl_2_, 200 µmol of each dNTP, 0.6 µmol of each primer and 50–100 ng of template DNA. The PCR conditions were: denaturation at 94°C for 4 min, followed by 35 cycles of 94°C for 1 min, 55°C for 1 min, 72°C for 1 min and a final extension for 10 min at 72°C. PCR products were separated in 6% denaturing polyacrylamide gels, 8% non-denaturing polyacrylamide gels or 1.5% agarose gels, depending upon the marker, visualized using silver staining [26] for polyacrylamide gels or ethidium bromide for agarose gels and photographed.

### Statistical Analysis and Genetic Linkage Map

Chi-squared analysis (χ^2^) was used to test agreement of expected and obtained segregation ratios. The genetic distances between markers and the *Yr26* locus were calculated with software JOINMAP version 4.0 [27] using the Kosambi mapping function [28] and a LOD score of 3.0 as a threshold. The genetic linkage map was drawn with the software Mapdraw V2.1 [29].

## Supporting Information

Figure S1 **Examples of PCR products amplified with four conserved markers.** CON-1 (a), CON-4 (b), CON-6 (c) and CON-7 (d); RP, 92R137; RB, resistant bulk; SP, AVS; SB, susceptible bulk; R, resistant plants; S, susceptible plants; Arrow indicated the polymorphic amplification products.
(TIF)

Table S1 **BLASTn search of** ***B. distachyon*** **and rice with ten mapped wheat ESTs.**
(DOC)
