# Supplementary figures and images for: Fine Mapping of Wheat Stripe Rust Resistance Gene Yr26 Based on Collinearity of Wheat with Brachypodium distachyon and Rice
Source: PLoS One. 2013 Mar 5;8(3):e57885. doi: 10.1371/journal.pone.0057885 (PMC3589488; doi:10.1371/journal.pone.0057885)

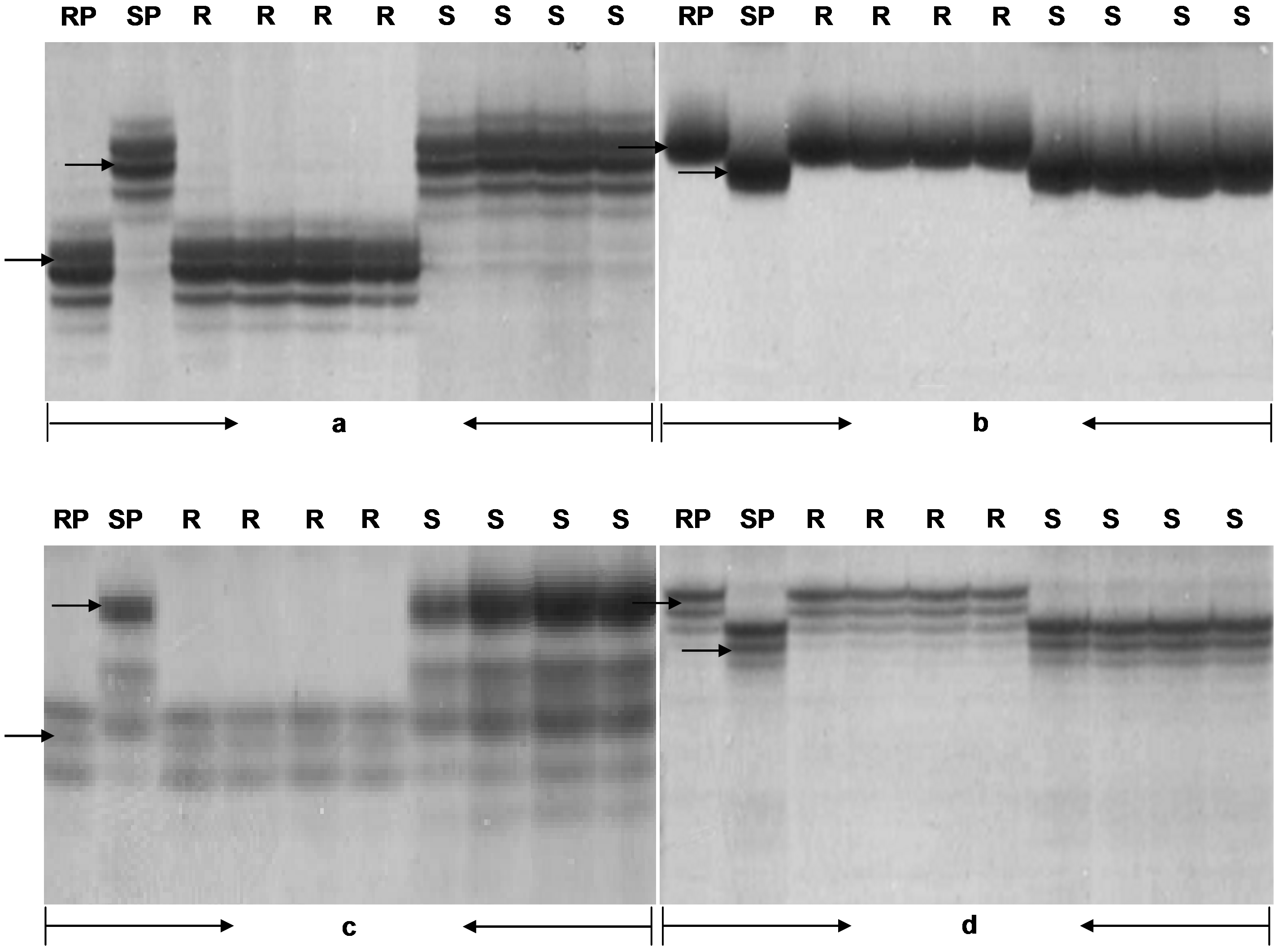

Supplement: Figure S1 — Examples of PCR products amplified with four conserved markers. CON-1 (a), CON-4 (b), CON-6 (c) and CON-7 (d); RP, 92R137; RB, resistant bulk; SP, AVS; SB, susceptible bulk; R, resistant plants; S, susceptible plants; Arrow indicated the polymorphic amplification products. (TIF) [file pone.0057885.s001.tif]
