# Supplementary material for: Fine Mapping of Wheat Stripe Rust Resistance Gene Yr26 Based on Collinearity of Wheat with Brachypodium distachyon and Rice
Source: PLoS One. 2013 Mar 5;8(3):e57885. doi: 10.1371/journal.pone.0057885 (PMC3589488; doi:10.1371/journal.pone.0057885)
Supplement: Table S1 — BLASTn search of B. distachyon and rice with ten mapped wheat ESTs. (DOC) [file pone.0057885.s002.doc]

**Table S1.** BLASTn search of *B.distachyon* and rice with ten mapped wheat ESTs

|  |  | *B. distachyon* | | | |  | Rice | | | |
| --- | --- | --- | --- | --- | --- | --- | --- | --- | --- | --- |
| Marker | EST | Chromosome | Score | Identity | E value |  | Chromosome | Score | Identity | E value |
| *STS-BQ5* | BQ160738 | 2 | 36 | 18/18(100%) | 0.73 |  | 10 | 36 | 18/18 (100%) | 1.3 |
| *STS-BQ6* | BQ165938 | 3 | 107 | 72/78 (92%) | 4e-22 |  | 10 | 78 | 64/71 (90%) | 6e-13 |
| *STS-CD28* | CD453471 | 3 | 165 | 101/107(94%) | 4e-39 |  | 10 | 115 | 73/78 (93%) | 6e-24 |
| *STS-BQ33* | BQ160383 | 3 | 502 | 331/357(92%) | e-140 |  | 10 | 509 | 332/357(92%) | e-143 |
| *STS-BE46* | BE493918 | 2 | 48 | 30/32 (93%) | 5e-04 |  | 11 | 38 | 22/23 (95%) | 0.83 |
| *STS-BE68* | BE443531 | 3 | 256 | 256/298(85%) | 9e-67 |  | 7 | 76 | 108/130(83%) | 3e-12 |
| *STS-BQ74* | BQ169964 | 1 | 165 | 122/135(90%) | 3e-39 |  | 2 | 121 | 91/101 (90%) | 8e-26 |
| *STS-CD77* | CD490549 | 2 | 153 | 143/165(86%) | 1e-35 |  | 5 | 147 | 149/174(85%) | 1e-33 |
| *WE201* | BE497109 | 3 | 40 | 20/20 (100%) | 0.084 |  | 9 | 103 | 127/152(83%) | 1e-20 |
| *WE173* | BF474347 | 5 | 40 | 23/24 (95%) | 0.12 |  | 8 | 38 | 19/19 (100%) | 0.9 |
